# Can the soil seed bank of *Rumex obtusifolius* in productive grasslands be explained by management and soil properties?

**DOI:** 10.1371/journal.pone.0286760

**Published:** 2023-06-02

**Authors:** Matthias Suter, Julie Klötzli, Deborah Beaumont, Aleš Kolmanič, Robert Leskovšek, Urs Schaffner, Jonathan Storkey, Andreas Lüscher

**Affiliations:** 1 Forage Production and Grassland Systems, Agroscope, Zurich, Switzerland; 2 Rothamsted Research, North Wyke, Okehampton, United Kingdom; 3 Agricultural Institute of Slovenia, Ljubljana, Slovenija; 4 CABI, Delémont, Switzerland; 5 Rothamsted Research, Harpenden, Hertfordshire, United Kingdom; University of Deusto: Universidad de Deusto, SPAIN

## Abstract

*Rumex obtusifolius* is a problematic weed in temperate grasslands worldwide as it decreases yield and nutritional value of forage. Because the species can recruit from the seed bank, we determined the effect of management and soil properties on the soil seed bank of *R*. *obtusifolius* in intensively managed, permanent grasslands in Switzerland (CH), Slovenia (SI), and United Kingdom (UK). Following a paired case-control design, soil cores were taken from the topsoil of grassland with a high density of *R*. *obtusifolius* plants (cases) and from nearby parcels with very low *R*. *obtusifolius* density (controls). Data on grassland management, soil nutrients, pH, soil texture, and density of *R*. *obtusifolius* plants were also collected. Seeds in the soil were germinated under optimal conditions in a glasshouse. The number of germinated seeds of *R*. *obtusifolius* in case parcels was 866 ±152 m^-2^ (CH, mean ±SE), 628 ±183 m^-2^ (SI), and 752 ±183 m^-2^ (UK), with no significant difference among countries. Densities in individual case parcels ranged from 0 up to approximately 3000 seeds m^-2^ (each country). Control parcels had significantly fewer seeds, with a mean of 51 ±18, 75 ±52, and 98 ±52 seeds m^-2^ in CH, SI, and UK, respectively, and a range between 0 and up to 1000 seeds m^-2^. Across countries, variables explaining variation in the soil seed bank of *R*. *obtusifolius* in case parcels were soil pH (negative relation), silt content (negative), land-use intensity (negative), and aboveground *R*. *obtusifolius* plant density (positive). Because a large soil seed bank can sustain grassland infestation with *R*. *obtusifolius*, management strategies to control the species should target the reduction in the density of mature plants, prevention of the species’ seed production and dispersal, as well as the regulation of the soil pH to a range optimal for forage production.

## Introduction

*Rumex obtusifolius* L. (broad-leaved dock) is a problematic perennial weed in grasslands of temperate regions worldwide as it decreases yield and the nutritional value of forage [1]. The species is an efficient resource competitor thanks to its root system with deep taproots [2] and, in managed grasslands, competes with the valuable forage species [3]. *Rumex obtusifolius* is able to flower several times annually, and large plants can produce up to 60,000 seeds in a single year [4]. Zaller [1] noted that many seeds just fall to the ground near the parent plant, and following Cavers and Harper [4] seed survival in *R*. *obtusifolius* can be up to 40 years, which suggests that the species can build a persistent seed bank (sensu Thompson et al. [5]) given sufficient opportunity for seed set.

Seeds of *R*. *obtusifolius* have high germination rates when exposed to light and fluctuating temperatures [6–8]. Regarding the dynamics of the soil seed bank of *R*. *obtusifolius*, this means that a large proportion of the seeds will germinate if they remain on or near the soil surface and have access to light [9]. Such conditions occur, for example, where *R*. *obtusifolius* plants produce seeds in regularly grazed or mown grasslands of temperate regions. In comparison, when seeds of *R*. *obtusifolius* are buried after dispersal and left undisturbed in the soil, they become dormant and can lead to seed banks with large numbers of viable seeds [9, 10].

Given the challenge of effectively controlling *R*. *obtusifolius* in managed grasslands [3, 11, 12], a large soil seed bank of the species can be a possible reason for the inefficiency of weed control methods. These generally aim to reduce established plants of *R*. *obtusifolius*, but do not directly target the input of seeds to the soil seed bank or the recruitment of seedlings from the seed bank [13]. Thus, in light of the longevity of *R*. *obtusifolius* seeds, populations might be able to continue to recruit seedlings from the seed bank despite measures that are effective against established plants. Data on the soil seed bank of *R*. *obtusifolius* could therefore give a better understanding of the species’ emergence potential in grasslands, which in turn could inform management practices to disrupt seed set and prevent or reduce re-establishment from the seed bank. An old report claims that, where the species is present in grassland, around 1200 seeds m^-2^ can be found in the soil [14]. However, no further information was given on the viability of these seeds and how the seed numbers were acquired, nor whether they vary according to the management and environmental conditions. Thus, data on the soil seed bank of *R*. *obtusifolius* are scarce.

So far, studies examining the influence of management and soil properties on seed banks of grassland species have focused on low-intensity and mesic systems, and *R*. *obtusifolius* remains largely unexplored [e.g., 15–17]. In a comprehensive survey, Bekker et al. [18] evaluated the soil seed banks of European grasslands and concluded that intensive agricultural management had a negative impact on seed banks [18]. Yet, land-use intensity may affect soil seed banks in complex ways, as management comprises several aspects, including mowing, grazing, and fertilisation [19], and each of them can affect seed banks in different ways [15, 17]. For example, in a field study of mesic grasslands, intensive mowing and fertilisation decreased seed bank species richness and density, probably by inhibiting seed set due to early cuts [17]. By contrast, grazing increased seed bank species richness, probably by allowing for a longer period of seed set for some species due to selective grazing and incomplete biomass removal [17]. Thus, both the type of management (mowing, grazing) and the frequency of defoliation will determine whether seed set is successful and a species is able to build up a soil seed bank. Moreover, past disturbance events and management efforts can have legacy effects on seed banks that can persist despite changes in aboveground vegetation [20, 21]. Generally, one can assume that a more intensive grassland management with higher defoliation frequencies diminishes the chances for seed production and thus, in the long term, should also keep the seed bank low.

In the study of Bekker et al. [18], which also included the measurement of soil properties (soil nutrients, organic matter, pH), only soil pH accounted for dissimilarities among seed banks. While there seems to be no direct pH effect on the germination of viable seeds in selected grassland species, pH can indirectly influence seed persistence by affecting soil microorganisms [22]. Low pH can hamper growth of soil fungi or bacteria [22, 23]. Because these microorganisms impact on seeds via lytic enzymes [24], seed damage should be reduced where pH is low, leading to more persistent seed banks [22]. In addition, soil texture may affect seed banks directly and indirectly. For example, in a three-year experiment seed burial by precipitation was slower and to lesser depths in clay soils than in sandy soils [25]. This implies that fewer seeds should naturally be incorporated into soils with increasing clay content, resulting in comparably smaller seed banks. Soil texture can also affect seed bank persistence indirectly through soil water: soils with smaller particle size have an increased water holding capacity and water retention potential [26], and (soil) water status generally influences seed persistence [27]. In comparison, soil nutrients seem to affect seed banks in grasslands only marginally [e.g., 18, 28, 29].

Here, we evaluate the soil seed bank of *R*. *obtusifolius* in intensively managed, permanent grasslands used for forage production in the three European countries Switzerland, Slovenia, and United Kingdom, covering a gradient of climate from Atlantic to continental. In each country, topsoil cores were taken from parcels with a high plant density of *R*. *obtusifolius* and from nearby parcels with a very low density of the species or no *R*. *obtusifolius* plants at all, and seeds of the samples were germinated in a glasshouse. In addition, we recorded data on management practice and analysed soil nutrients and texture. We hypothesised that i) *R*. *obtusifolius* builds a seed bank in the topsoil of managed, permanent grasslands, where the species has been abundant for several years, ii) increased management intensity with higher defoliation frequencies diminishes the seed bank of *R*. *obtusifolius*, and iii) soils with lower pH have higher *R*. *obtusifolius* seed banks, while soils with smaller particle size have smaller *R*. *obtusifolius* seed banks. To the best of our knowledge, this is the first time that the seed bank of *R*. *obtusifolius* in permanent forage grassland has been quantified and related to management and soil properties.

## Materials and methods

### Selection of parcels and management data

The study was conducted in Switzerland (CH hereafter), Slovenia (SI), and United Kingdom (UK) following a common protocol. The selection of parcels was restricted to intensively managed, permanent grasslands, i.e., grasslands under medium to high management intensity used for forage production and established for at least five years; the majority of parcels had been managed as grasslands for more than twenty years. All grassland was regularly mown and/or grazed and was dominated by grass species (57–73%) with substantial amounts of legumes (12–16%) and forbs (13–31%) (ranges across countries). In CH, the sampling area covered the central Plateau, the Jura region and the Northern Prealps. In SI, the area covered the central Osrednja/Jugovzhodna Slovenija regions and the Podravje/Pomurje regions in the North East, and in UK, the area covered the South West region, with farms predominately being located in Devon but expanding into east Cornwall (see S1 Table for further characteristics of the grasslands).

Parcels with populations of *R*. *obtusifolius* were identified with support from the agricultural advisory services. The sampling of parcels followed a paired case-control study [30], a sampling design that has successfully been used to evaluate the occurrence of two *Senecio* species [31, 32]. At each location, two parcels were selected: one with at least one *R*. *obtusifolius* plant m^-2^ (case) and a nearby parcel with a maximum of four *R*. *obtusifolius* plants 100 m^-2^ (control). The distance between pairs of parcels was generally less than one km (mostly < 600 m), but was occasionally larger if no matching control parcel was found nearby. This sampling regime resulted in similar environmental conditions regarding each of temperature and precipitation, while slope and exposition of the pairs of parcels were selected to be as similar as possible. Control parcels that had been treated with herbicides against *R*. *obtusifolius* within the last five years were excluded. All parcels were private land owned by farmers, and we were given verbal assurance to take measurements on the fields. In total, 80, 40, and 36 parcels were selected in CH, SI, and UK, respectively.

Data on the management of parcels were acquired through farmer interviews. The type of management (mowing, mixed mowing-grazing, rotational or continuous grazing), the number of mowing and/or grazing events per year, and the amount of applied N fertilisers was recorded. Data on N applied, mowing, and grazing intensity were used to calculate the quantitative, continuous index of land-use intensity (LUI) following Blüthgen et al. [19] (see S1 Appendix for details on the calculation of LUI). Moreover, information about the parcel’s history (time horizon over the last ten years) was recorded, which included disturbance events (e.g., drought event, intensive livestock trampling, heavy machinery, construction of drainage tubes, strong impact of mice or boars) and regulation methods applied on *R*. *obtusifolius* plants (e.g., application of herbicides, steam treatment, pulling/digging, cutting of aboveground biomass; see S2 Table for means of management variables).

### *Rumex obtusifolius* density and soil sampling

Within a radius of 10 m from the center of each selected parcel (case, control), two measurement plots (dimension 3 m × 3 m) were identified. In each measurement plot, the plant density of *R*. *obtusifolius* was determined. A plant individual was recorded on the basis of having a distinct rosette (established plants) or having at least one fully developed leaf (seedlings). To test for seed germination, samples from the topsoil (0–10 cm) were cored with a soil auger (15–21 cores per measurement plot, depending on country) so that a defined soil volume was collected (ca. 0.7 l per measurement plot), which allowed the number of germinated seeds of *R*. *obtusifolius* per m^2^ to be determined. A similar number of separate soil cores were taken for the analysis of soil pH, nutrients and texture (10 cm deep). For both type of sample (seed germination, soil analysis), individual cores from the two measurement plots were bulked resulting in one sample per parcel, and all material was stored in plastic bags at 4°C under dark conditions until further processing.

### Germination procedure

**Fig 1:**
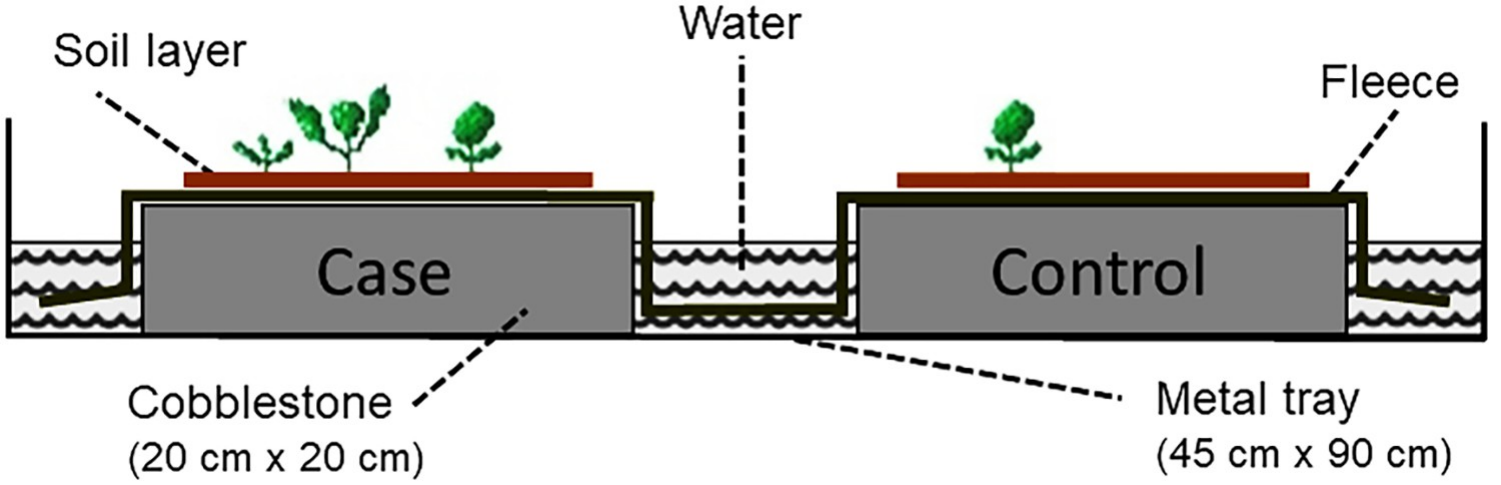
Technical facility at Agroscope, Zürich, for the seed germination test to ensure constant moisture conditions to the soil layers and the seeds. Figure adapted from Suter and Lüscher [34]. The large metal tray was made of galvanized steel. The same device was used at Rothamsted Research, Harpenden, with slightly different dimensions.

Soil samples to test for seed germination were prepared following ter Heerdt et al. [33]. The soil was concentrated with a jet of water through a coarse (5 mm mesh width) and fine sieve (0.5 mm) to remove stones, large roots, vegetative parts, and very fine soil particles. The fine mesh was small enough to retain all seeds of *R*. *obtusifolius*. The germination procedure proposed by ter Heerdt et al. [33] was further improved. To ensure constant moisture during germination, a modified version of the device proposed by Suter and Lüscher [34] was used. Two cobblestones (dimension 20 cm × 20 cm) were placed in a metal tray (dimension 45 cm × 90 cm) and were covered by a fleece, which was submerged into water filled into the tray (Fig 1). The concentrated soil of a pair of parcels was each spread on the fleece on a surface area of 18 cm x 18 cm in one tray, thereby creating layers of approximately 5 mm thickness.

The germination test for CH and SI samples was performed in a glasshouse at Agroscope, Zürich. Germination was recorded three times a week. Seedlings of *R*. *obtusifolius* were counted and removed when the first fully developed leaf was identified; other seedlings of grass and forb species were removed once they were identified as non-*R*. *obtusifolius* species. The soil layer was thoroughly crumbled at day 21, 35 and 49 after set-up to optimise light exposure to all seeds. Germination was evaluated for 63 days; no or only a marginal germination was observed subsequent to this period. The seed bank samples from the UK were germinated in a glasshouse at Rothamsted Research, Harpenden. Assessments were impacted by Covid-19 restrictions: seedlings were assessed three times and the soil crumbled once. Because the seedlings were larger, it was possible to identify all emerged broad-leaf species on the assessment dates (see S1 Appendix for further details on the germination procedure).

### Analysis for soil nutrients and soil texture

All samples for the soil nutrients and texture analyses were first dried to constant weight (60° C max) and then sent to CH, where they were analysed at Agroscope, Zürich. The analysis followed standard methods [35]. Soil nutrients analysed included phosphorus (P), potassium (K), magnesium (Mg), and calcium (Ca). Contents of P, K, Mg, and Ca were determined using extractions with ammonium acetate and ethylene-diamine-tetraacetic acid (EDTA) (see S1 Appendix for details on these analyses, incl. pH and soil texture).

### Data analysis

The number of germinated seeds of *R*. *obtusifolius* in case and control parcels at the end of the experiment was analysed with a generalized linear mixed-effects model. Numbers of seeds (scaled per m^2^) was modelled as a function of ‘parcel type’ (factor with two levels: case, control), ‘country’ (factor with three levels: CH, SI, UK), and their interaction. A random intercept was specified for pairs of case and control parcels at a site. We had to imply a zero-inflated negative binomial hurdle model (ZINBH) because preliminary analyses revealed substantial zero-inflation and overdispersion of the data (see S1 Appendix, for details on the ZINBH).

To further explain variation in the soil seed bank of *R*. *obtusifolius*, the final number of germinated seeds in case parcels was regressed on the management and soil variables (S2 Table) and *R*. *obtusifolius* plant density while always including ‘country’ as a factor. ‘Management type’ was coded as a factor with four levels (mowing, mixed mowing-grazing, rotational grazing, continuous grazing), while ‘disturbance’ and ‘regulation’ were each coded as factors with two levels (yes, no). A generalised linear model (GLM) implying the negative binomial distribution and a log-link was used, and forward selection under the second-order Akaike Information Criterion was used to justify the inclusion of terms in the model (AICc, [36]). Interactions between each of the finally selected variables and the factor ‘country’ were tested. Only case parcels were used for this regression because the density of *R*. *obtusifolius* plants in control parcels was restricted by design and the observed number of seeds in these parcels was mostly zero or low (see below). Thus, the range of seeds in control parcels was too small to allow for a reasonable regression on predictor variables. Both of these analyses (ZINBH, GLM) were done using the statistics software R, version 4.2.1 [37] and the package glmmTMB for the ZINBH model [38].

Finally, a Redundancy Analysis (RDA) of the seed bank communities assessed in the UK was done using the totals from all assessment dates. Grass species were excluded from the analysis (not identified to species) as were any species that only occurred in a single sample. The analysis included parcel type (case, control) as a categorical explanatory variable and was done using Canoco 5 [39]. This RDA was performed only at UK because only here, due to unforeseen Covid-19 restrictions and limited access to the experiment, seedlings were so large at the time of assessment that emerging broad-leaf species could be determined. This was not planned at the other two countries.

## Results

**Fig 2:**
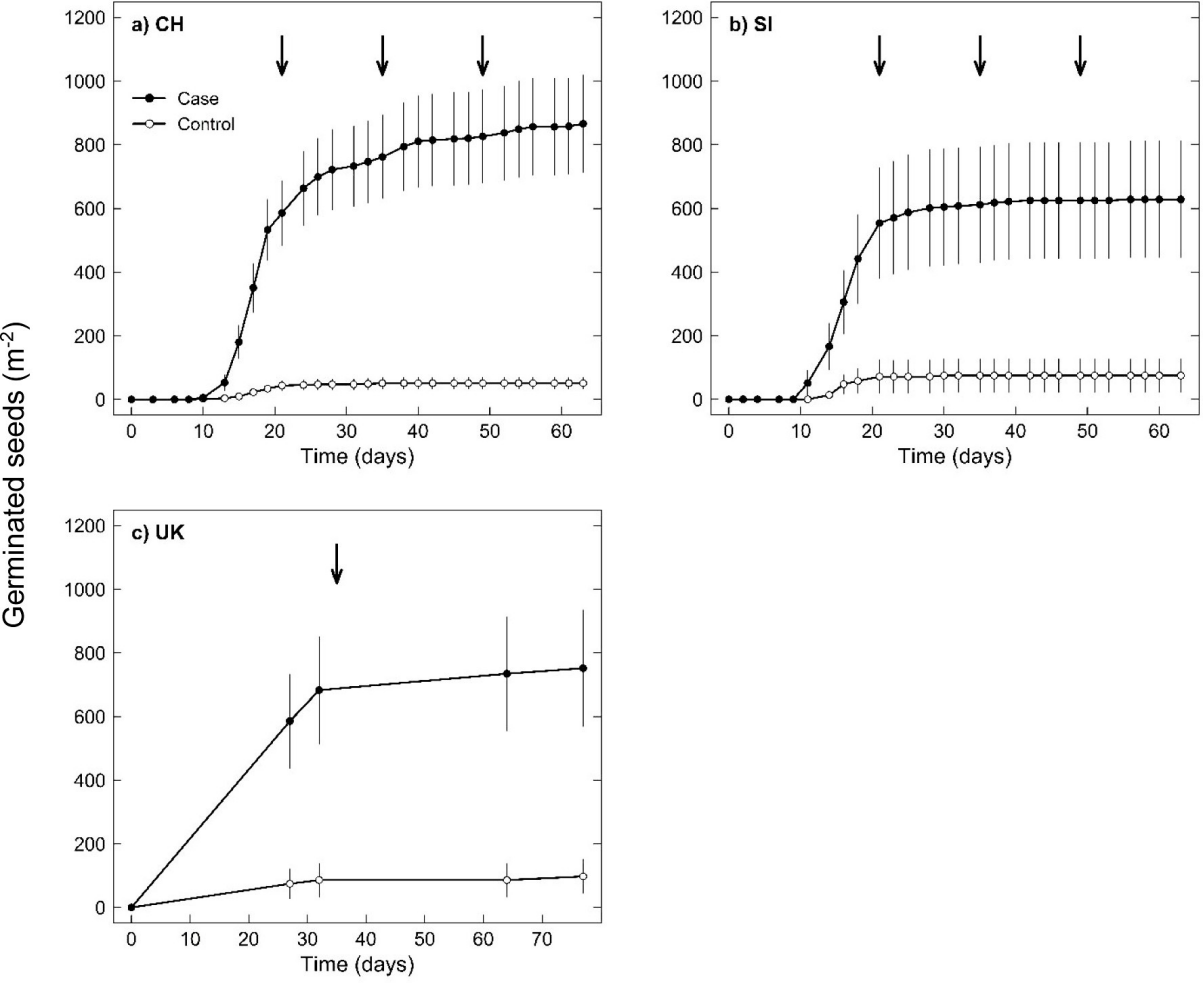
Germinated seeds of *Rumex obtusifolius* from grasslands with high density of the plant (case: ≥ 1 plant m^-2^) and nearby parcels with low density or no plants of *R*. *obtusifolius* at all (control: ≤ 4 plants 100 m^-2^). Displayed are means ±1 SE from Switzerland (CH) (a), Slovenia (SI) (b), and United Kingdom (UK) (c). At noted dates (↓) the substrate was thoroughly crumbled to optimise light exposure to all seeds. At UK due to Covid-19 restrictions, access to the experiment was allowed for only 3 times over the planned duration of 63 days, and the soil substrate was crumbled only once. The experiment, however, was run for another 14 days.

**Fig 3:**
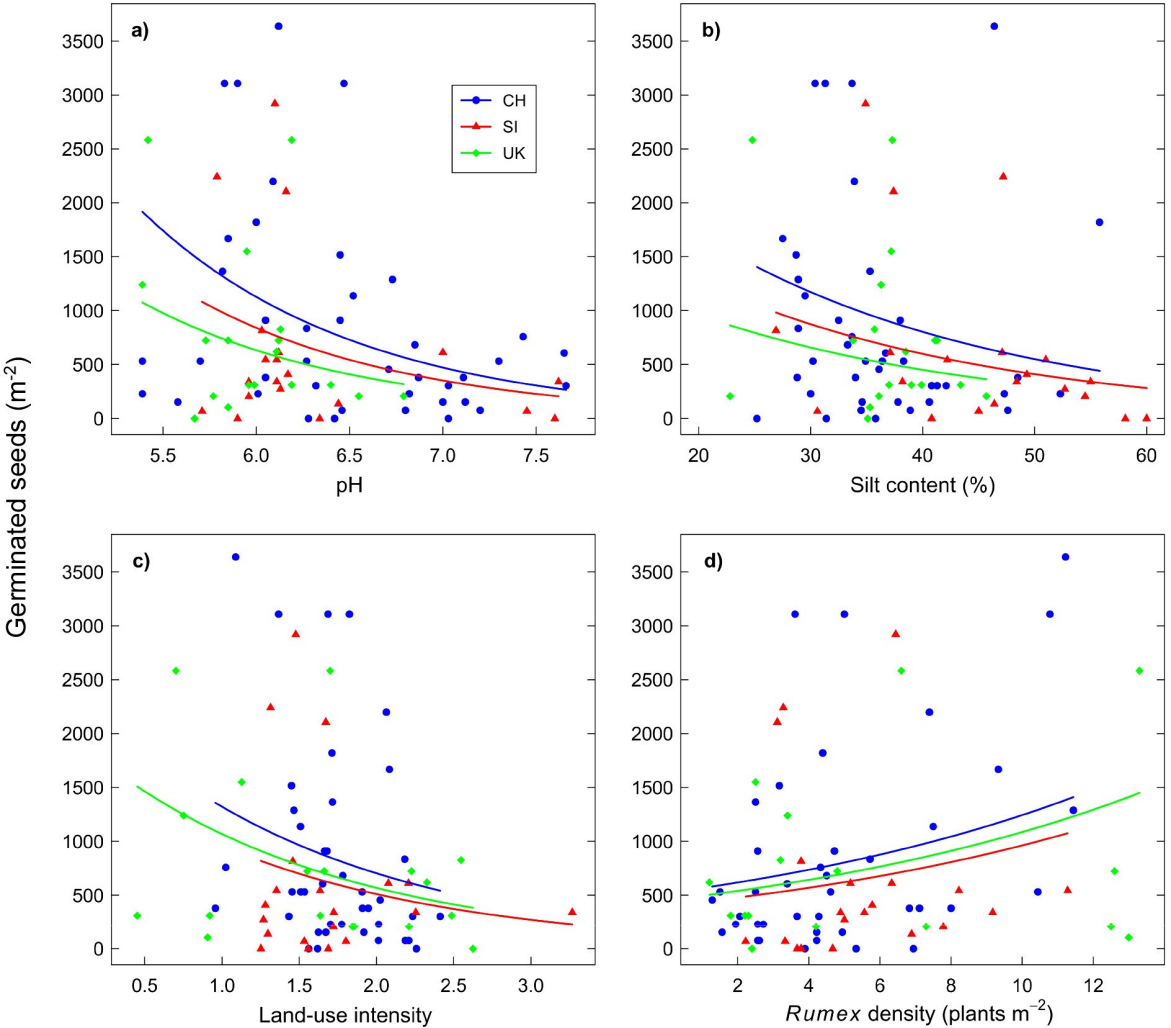
Germinated seeds of *Rumex obtusifolius* in case parcels depending on soil pH (a), soil silt content (b), land-use intensity (c), and density of *R*. *obtusifolius* plants (d) in the three countries Switzerland (CH), Slovenia (SI), and United Kingdom (UK). Estimated lines are based on generalised linear models and are displayed at the mean of each other predictor. In panel d) one outlier in UK with 26 *R*. *obtusifolius* plants m^-2^ was omitted; inclusion would have resulted in qualitatively same results.

The number of germinated seeds of *R*. *obtusifolius* in case parcels was 866 ±152 m^-2^ (CH, mean ±SE), 628 ±183 m^-2^ (SI), and 752 ±183 m^-2^ (UK) (Fig 2), with no significant difference among countries (Table 1: p = 0.746). Yet, seed numbers at the level of individual case parcels ranged greatly from 0 up to approximately 3000 seeds m^-2^ (Fig 3, each country). In each country, control parcels had significantly fewer seeds (Fig 2, Table 1: p < 0.001), with a mean of 51 ±18, 75 ±52, and 98 ±52 seeds m^-2^ in CH, SI, and UK, respectively, and a range between 0 and up to 1000 seeds m^-2^. The great majority of control parcels had no seeds of *R*. *obtusifolius* at all; the respective proportions of controls with no *R*. *obtusifolius* seeds were 0.78 (CH), 0.85 (SI), and 0.67 (UK). Yet, although 13% of the control parcels had no established *R*. *obtusifolius* plants, a *R*. *obtusifolius* seed bank between 76 and 930 seeds m^-2^ was still observed.

**Table 1:** Summary of analysis for the effects of *Rumex obtusifolius* plant occurrence (parcel type: case, control) and country (CH, SI, UK) on the number of germinated seeds.

Variable	df	χ^2^	p
Parcel type	1	14.59	< 0.001
Country	2	0.59	0.746
Parcel type × Country	2	0.88	0.644

*R*. *obtusifolius* plant density in case parcels was ≥ 1 plant m^-2^, while it was ≤ 4 plants100 m^-2^ in control parcels.

Regarding the mean emergence time in CH and SI, the first seedlings of *R*. *obtusifolius* with at least one fully developed leaf were observed around ten days after onset of testing (Fig 2). Because the UK data had fewer assessment dates, it was not possible to calculate a comparable rate of germination. Moreover after 32 days, 85%, 97%, and 91% of the finally germinated seeds from case parcels had germinated in CH, SI, and UK samples, respectively, and only little germination occurred thereafter (Fig 2).

Variables affecting the number of germinated seeds of *R*. *obtusifolius* in case parcels were soil pH and silt content: soils with higher pH (significant) and higher silt content (marginally significant) had fewer seeds (Table 2, Fig 3). With two exceptions, none of the other variables (S2 Table) had an effect on seed numbers, neither when tested in addition to country, pH, and silt content (no improvement of the model based on the AICc), nor when tested alone given country (all p > 0.1, mostly p > 0.3). The two exceptions were LUI and *R*. *obtusifolius* plant density, both of which had a marginal effect on seeds of *R*. *obtusifolius* when tested alone. Land-use intensity negatively affected seed numbers (χ^2^ = 3.54, df = 1, p = 0.060), while *R*. *obtusifolius* plant density was positively related to seed numbers (χ^2^ = 3.18, df = 1, p = 0.075, Fig 3). Interactions between the factor ‘country’ and either pH (χ^2^ = 0.30, p = 0.862), silt content (χ^2^ = 3.67, p = 0.160), LUI (χ^2^ = 0.58, p = 0.747), or *R*. *obtusifolius* plant density (χ^2^ = 1.77, p = 0.414) were not significant (all df = 2), meaning that effects were comparable across countries. Also, the four variables pH, silt content, LUI, and *R*. *obtusifolius* plant density were not or only weakly correlated to each other (S1 Fig), indicating that they affected seed numbers largely independently. Notably, the type of management (S2 Table) did not affect the seed bank of *R*. *obtusifolius* (χ^2^ = 1.83, df = 3, p = 0.608), nor did any of the soil nutrient variables (see S2 Table for means and standard deviations of all soil variables). Finally, the overall *R*^2^ of the regression model, which included country, pH, and silt content, was 0.28 (Table 2).

**Table 2:** Summary of analysis for the effects of country and soil variables on germinated seeds of *Rumex obtusifolius* in case parcels at the three countries CH, SI, and UK.

Variable	Df	χ^2^	p	ΔAICc^a^
Country	2	0.76	0.690	-
pH	1	7.53	0.006	-5.2
Silt content	1	3.49	0.062	-1.1

Terms added sequentially (first to last). Only variables that lowered the AICc upon inclusion in the model are presented; see S2 Table for all tested variables. The R^2^ of the model was 0.28 (following Nakagawa et al. [40]). See S3 Table for the regression coefficients and their confidence intervals.

^a^Change of the AICc by inclusion of variable.

## Discussion

### Soil and management factors affecting the seed bank of *R*. *obtusifolius*

We found, in all three countries, comparably high numbers of viable seeds of *R*. *obtusifolius* in managed, productive grasslands that were infested with the species. At ~750 seeds m^-2^ (approx. mean across the three countries) the seed bank of *R*. *obtusifolius* appears to be high compared to the seed bank of other grassland species of temperate climates [15, 20, 41]. For example in calcareous grasslands, seed banks of individual species were generally less than 100 seeds m^-2^ [15, 20, 28]. And in grasslands under low-intensity management, only a few species had seed banks larger than 700 seeds m^-2^, while the majority had seed numbers lower than 400 seeds m^-2^ [29]. At first glance, a large seed bank and effective germination in response to light appears contradictory. In regularly defoliated grasslands, seed banks may be depleted due to repeated disturbance of the soil surface by grazing livestock or intensive cutting regimes, providing light to seeds and allowing for germination [9, 42]. However, fresh ripe seeds of *R*. *obtusifolius* have innate dormancy [43] and a fraction of these seeds can become covered by litter and/or be buried in the topsoil by precipitation [25] or the activity of mice, earthworms or livestock [44, page 137]. Because a soil cover of only 2–3 cm strongly reduces germination in *R*. *obtusifolius* [9, 10], over time these seeds accumulate in the soil and can form a persistent seed bank [5]. Given the low proportion of seeds that remain viable at the soil surface or the topmost soil layer [45], our results imply that in the grasslands included in our study *R*. *obtusifolius* must have repeatedly formed seeds in the past, including the preceding five years prior to investigation.

In case parcels we generally observed a large seed bank of *R*. *obtusifolius* on average, but also a large variation in seed numbers (Fig 3). Regarding the factors that were found to affect this variation, pH had the strongest influence (Table 2: compare ΔAICc). Basto et al. [22] have demonstrated an indirect influence of pH on seed persistence in the soil via soil microorganism. Soil fungi, for example, impact on seeds via lytic enzymes that break down the substrate [24]. Where growth of soil fungi or bacteria is hampered by low pH [22, 23], seed attack and membrane damage are diminished, which can result in a higher proportion of persistent seeds in soils. Our results, which reveal increased numbers of *R*. *obtusifolius* seeds from soils with a pH < 6.0–6.5, are in agreement with this explanation. Basto et al. [22] found a very similar pattern between seed bank size and pH in selected grassland species other than *R*. *obtusifolius*, although in their study higher seed numbers coincided with a pH below 5.6. When they applied fungicide, seed persistence in soils with pH > 5.6 was increased in the three species tested, suggesting that soil microorganisms (fungi) caused physical damage to seeds. In terms of practical management recommendations, a pH between 5.9 and 7.2 is recommended for optimal forage production [46]. Thus, keeping the pH in this range should also contribute to the regulation of the soil seed bank of *R*. *obtusifolius*.

Soil texture had a weak influence on the number of germinated seeds of *R*. *obtusifolius* (Table 2). Our data support the concept of mechanical hindrance of seed incorporation into soils with smaller particle size [25], as soils with higher silt content had a tendency for smaller seed banks (Fig 3B). There may have also been an indirect effect of higher silt content on seed bank persistency through increased water retention potential [26], which may have promoted water-saturated conditions. Yet, soil water status could not reasonably be recorded over longer periods in our field study on managed grasslands; thus, any indirect effect of silt content on seed persistence of *R*. *obtusifolius* remains uncertain.

Land-use intensity had a negative impact on the seed bank of *R*. *obtusifolius*, in agreement with previous work on other species in low-intensive grasslands [17, 18, 41]. The effect of LUI in our study was marginal; yet, given that all our parcels were under medium to high management intensity, we presume LUI affected seed banks more if a larger range of management intensities were investigated. Notably, LUI was not correlated to *R*. *obtusifolius* plant density (S1 Fig), which suggests that a more intensive management mainly hindered the species’ seed production and thus input into the seed bank, but did not consistently affect the abundance of mature *R*. *obtusifolius* plants once established. This seems to contradict studies that have found an increasing similarity between seed bank and established vegetation due to management practice [15, 16]. Yet, established *R*. *obtusifolius* plants are hard to control by cutting alone [3, 11] because the species has a strong re-growth potential. Its taproots serve to store carbohydrates [47] and the root system allows for clonal growth [2]. We conclude that frequent defoliation of grasslands can contribute to prevent the species’ seed formation and keep the seed bank low, an effect that could also be achieved by specific cutting of flowering stems. A direct effect of regulation interventions on the seed bank of *R*. *obtusifolius* could not be demonstrated here because all case parcels in SI and UK had some kind of weed control, and the lack of variation in a predictor renders any analysis impossible. In CH, the majority of case (and control) parcels were regulated (S2 Table), but no effect of measures on the seed bank was found.

In comparison, we found control parcels where *R*. *obtusifolius* plants were not present at all, but where the species had a seed bank of up to 1000 seeds m^-2^. There is evidence that past disturbance events allowing for seed production of weed species can persist in an augmented soil seed bank, although the event being no more detectable in aboveground vegetation after only two years [21]. In our study, the results of the community analysis of the UK seed bank samples (S2 Fig) support the hypothesis that seed banks may be a legacy of previous infestation, as seed survival in *R*. *obtusifolius* can be up to 40 years [4]. *Rumex obtusifolius* was particularly associated with annual species that are typical of disturbed habitats (including *Chenopodium album* L. and *Capsella bursa-pastoris* (L.) Medik., S2 Fig), reflecting a previous disturbance event that allowed *R*. *obtusifolius* to establish and return large quantities of seed. Previous authors have also highlighted the association of *R*. *obtusifolius* with annual species and it has been described as an ‘interloper among arable weeds’ [4]. The control parcels with a seed bank also demonstrate that grassland can be managed so that germination of *R*. *obtusifolius* seeds is avoided or minimised, most probably through avoidance of soil disturbance.

The relationship between established *R*. *obtusifolius* plant density and the seed bank was evident in two ways. First, the significant difference in seed numbers between control and case parcels (Fig 2, Table 1) indicates a clear association, as plant density was restricted by design to ≤ 4 plants 100 m^-2^ in control parcels, but ranged between one and more than 12 plants m^-2^ in cases (Fig 3D). Second, variation in the soil seed bank of case parcels could partly be explained by the density of established *R*. *obtusifolius* plants (Fig 3D). We acknowledge that this study cannot demonstrate causality and it remains open whether high plant density resulted in the high seed bank or vice versa. However, the relationship allows for the possibility of sustained infestation of grasslands through seedling recruitment from the soil seed bank given that conditions for germination are favourable. For example, if we assume a germination rate of 20% of existing seeds and no further seed production and death, a seed bank of 750 germinable seeds m^-2^ would need about 20 years to deplete down to 8 seeds m^-2^. Thus, where *R*. *obtusifolius* has built up a soil seed bank, it must be assumed that seedlings can emerge for many years—the prevention of seed formation should, therefore, be given a high priority to control the species.

The soil variables selected into the final model explained about 30% in the variation of germinated seeds of *R*. *obtusifolius* in the investigated grasslands (Table 2), and adding LUI and *R*. *obtusifolius* plant density to the model would not have increased this value. A large variation in soil seed banks of grasslands is not uncommon [29, 34, 41]. Thus, in this context explaining 30% of the variance in our data was a useful result, given that we did not account for other factors. For example, seeds of *R*. *obtusifolius* can be imported to the field with slurry or manure [48, 49], potentially enriching the seed bank irrespective of any other influence. Most importantly, super spreader, stochastic events by single plants can be an important reason for seed bank variation. If 5–6 plants (mean m^-2^ across the three countries) are allowed to produce up to 60,000 seeds per plant [4], this will potentially result in a seed set of 330,000 seeds. With such an event occurring only once or twice during the last 5–40 years, this would result in considerably higher seed numbers without an explanation, because these events were most probably not recorded. Other factors that can affect seed banks are soil temperature and N-P-K fertilisers [27, 28, 50]. Germination of *R*. *obtusifolius* is strongly promoted by diurnal temperature cycles that occur in early spring and autumn in temperate regions [51]. With one sampling event per site, we did not measure soil temperature, and none of the analysed soil nutrients explained variation in the number of germinated seeds in agreement with other studies [18, 29, 52]. Thus, other factors affecting the soil seed bank of *R*. *obtusifolius* remain to be identified.

### Methodological aspects

The selection of our case parcels was based on information from the agricultural advisory services in each country and did not follow a random sampling procedure. In doing so, we followed previous survey studies focusing on seed banks of specific grassland types [17, 18, 29]. This, however, means that we cannot provide results about the frequency of occurrence of *R*. *obtusifolius* and the linkage to its seed bank in general, but that our findings are relevant to typical situations of intensively managed, permanent grasslands highly infested with the species. The fact that the seed bank of *R*. *obtusifolius* was of comparable size and was affected by the same management and soil drivers in the three countries gives our results a general relevance.

The germination method proposed by ter Heerdt et al. [33] has been shown to effectively break dormancy, and in our study the germination device (Fig 1) ensured constant moisture levels in the concentrated soil layer. Moreover, the diurnal cycles of light and temperature in the glasshouses during the tests were within the optimal conditions for the germination of seeds of *R*. *obtusifolius* [6], and the substrate was crumbled to optimise light exposure to all seeds. Under such conditions close to 100% of *R*. *obtusifolius* seeds will germinate [6]. We did not determine the exact germination percentages. Determining germination percentages is generally done in laboratory tests, where a defined number of seeds is germinated (usually in dishes on filter paper, [e.g., 7, 43]). However, such a task is hardly feasible with soil samples, and if done comes with high uncertainty. Thus, because there was hardly any germination after 60 days (Fig 2), we are confident that the size of the seed bank of *R*. *obtusifolius* as reported here provides a reliable estimate about the situation in managed, productive grasslands infested with the species.

### Implications for management

The occurrence of *R*. *obtusifolius* in grasslands has been associated with poor management [1]. Although a specific management intervention may not change the seed bank of *R*. *obtusifolius* in the short-term, an adapted management practice can do so in the mid- to long-term. Here, we have revealed several factors that acted over longer time scales and that partly can explain seed bank variation of *R*. *obtusifolius* in productive grasslands, and three of them were related to management. Because large seed banks of *R*. *obtusifolius* can result in continuous seedling recruitment and thus permanent infestation of grasslands, we claim that management strategies to control the species in grasslands should also aim at regulating soil pH to a range optimal for forage production, lowering the density of mature plants, and preventing the species’ seed production and dispersal.

## Supporting information

S1 Appendix Supporting information on germination conditions, analyses of soil nutrients and data analysis. (PDF)

S1 Table General grassland characteristics at the three countries Switzerland (CH), Slovenia (SI), and United Kingdom (UK), where parcels with high and very low density of *Rumex obtusifolius* were investigated to determine the soil seed bank of the species. (PDF)

S2 Table Mean (SD) or frequency of management and soil variables for parcels with high density of *Rumex obtusifolius* (Case) and parcels with very low density or no plants of the species (Control) in the three countries Switzerland (CH), Slovenia (SI), and United Kingdom (UK). (PDF)

S3 Table Regression coefficients of variables affecting the number of germinated seeds of *Rumex obtusifolius* in case parcels at the three countries Switzerland (CH), Slovenia (SI), and United Kingdom (UK). (PDF)

S1 Fig Correlation of variables affecting the number of germinated seeds of *Rumex obtusifolius* in case parcels in the three countries Switzerland (CH), Slovenia (SI), and United Kingdom (UK). Lower triangle shows Pearson’s coefficients of partial correlation, given country. None of the correlations was significant (p > 0.40 each, with the exception of pH versus land-use intensity: p = 0.16). One outlier in UK with 26 *R*. *obtusifolius* plants m^-2^ was omitted; inclusion would have resulted in qualitatively same results. See S2 Table for units of variables.
(JPG)

S2 Fig Results of UK seed bank analysis including data on all germinated species presented as a Redundancy Analysis with only the ten most dominant species included. *Rumex obtusifolius* (Rumob) was associated with a more diverse seed bank dominated by annuals. CASE: ≥ 1 *R*. *obtusifolius* plant m^-2^, CONTROL: ≤ 100 *R*. *obtusifolius* plants m^-2^, Capbp: *Capsella bursa-pastoris*, Cerfo: *Cerastium fontanum*, Cheal: *Chenopodium album*, Chepo: *Chenopodium polyspermum*, Cirvu: *Cirsium vulgare*, Matre: *Matricaria recutita*, Perla: *Persicaria lapathifolia*, Plama: *Plantago major*, Ranre: *Ranunculus repens*.
(JPG)
