# Supplementary material for: Can the soil seed bank of Rumex obtusifolius in productive grasslands be explained by management and soil properties?
Source: PLoS One. 2023 Jun 2;18(6):e0286760. doi: 10.1371/journal.pone.0286760 (PMC10237634; doi:10.1371/journal.pone.0286760)
Supplement: S1 Appendix — (PDF) [file pone.0286760.s001.pdf]

S1 Appendix. Supporting information on germination conditions, analyses of soil nutrients and data analysis

Land-use intensity

Information received from the farmers about the duration of grazing periods and the number of livestock allowed to calculated the grazing intensity as livestock unit days of grazing ha^{-1} year^{-1}. The amount of plant-available nitrogen (N) applied per year was calculated for the different forms of mineral and organic fertilisers, following standard tables of the three countries (Richner et al., 2017; Mihelič et al., 2010; AHDB, 2021). Data on plant-available N applied, mowing, and grazing intensity was then used to calculate the quantitative, continuous index of land-use intensity (LUI) following Blüthgen et al. (2012). Because we had to assume differing management intensities among the three countries due to differing pedo-climatic conditions, the LUI index was standardised following Blüthgen et al. (2012) with the respective country means of the three components of the index: fertilisation (plant-available nitrogen applied), mowing (number of mowing events), and grazing intensity (livestock unit days of grazing ha^{-1} year^{-1}). As suggested by Blüthgen et al. (2012), we applied the square-root transformation on the LUI to achieve a more even distribution. These values are presented in S2 Table.

Germination conditions

The germination test for CH and SI samples took place in a shaded glasshouse of Agroscope, Zürich, that had light (natural and supplementary) between 06:00 and 22:00 (16h/8h diurnal cycles). Temperature were generally maintained at 24 °C during daytime and at 18 °C during night, but temperatures sometimes rose to 31 °C during warm days. These conditions were within the optimal conditions for the germination of seeds of *R. obtusifolius* (Totterdell & Roberts, 1980).

The seed bank samples from the UK were germinated in a glasshouse at Rothamsted Research in the months of March, April and May, set up to replicate the conditions at Agroscope except that supplementary lighting was not used because of high natural light levels. Average daytime temperatures (06:00-22:00) were 25.5 °C and night time temperatures 18.9 °C. The experimental set-up was the same as described in the main text. Due to the pandemic situation,

access to the experiment was allowed for only 3 times over the planned time of 63 days and the soil substrate was crumbled only once. The experiment, however, was run for another 14 days.

Analysis of soil nutrients and soil texture

Soil samples were first re-dried to 40 °C and sieved through a 2 mm mesh before analysis. Concentrations of phosphorus (P), potassium (K), magnesium (Mg), and calcium (Ca) were determined using extractions with ammonium acetate and ethylene-diamine-tetraacetic acid (EDTA). 10 g soil was extracted for 1 h in 100 mL of a solution of 0.5 M ammonium acetate, 0.5 M acetic acid and 0.02 M EDTA at pH 4.65 (temperature 23 ± 1 °C). After 1 h paper filtration, K, Mg and Ca concentration are determined by ICP-OES (inductively coupled plasma-optical emission spectroscopy).

The pH was determined by mixing one part of soil with 2.5 parts of distilled water. The mixture was equilibrated between 12 and 18 hours before measurement.

Organic carbon (C-org) was determined with potassium dichromate in sulphuric acid, which oxidizes the organic carbon to CO_2. Back-titration of the remaining dichromate with Fe^{2+} allows the calculation of C-org (see Agroscope, 2020 for details). Percentage values of clay and silt were determined by sedimentation, while percentage sand was calculated as the difference between the sum of the first three parameters (C-org × 1.725, clay, silt) and 100%.

Data analysis

The number of germinated seeds of *R. obtusifolius* at the end of the germination experiment was analysed with a zero-inflated negative binomial hurdle model (ZINBH). ZINBH models have two components: a first component models the zero *versus* the non-zero counts, and a second component – here a truncated negative binomial model – is employed for the non-zero counts. Let S_i denote the cumulative number of germinated seeds in a soil sample at the end of the germination test. Then, if $S_i = 0$:

$S \sim \mathit{Binomial}(\pi)$

$\Pr(S_i = 0) = \pi_i$

$\mathrm{logit}(\pi) = \gamma_1 + \beta_1 \mathrm{Par\_Type}$

with parcel type (Par_Type) being a factor of two levels (0 for control parcels, 1 for case parcels). The analysis revealed that only the variable Parc_Type had a relevant effect on π, but not country.

If $S_i > 0$:

S ~ *Truncated NB*(μ,α), with α being a scaling parameter

$$E(S) = (1 - \pi)\frac{\mu}{1 - (1 + \alpha\mu)^{-1/\alpha}}$$

$$\log(\mu) = \gamma_2 + \beta_2 Par\_Type_i + \beta_3 Country_i + \beta_4 Parc\_Type \times Country + \lambda Tray$$

where 'Country' is a factor with three levels (CH, SI, UK) and λ is a random parameter with $\lambda \sim N(0, \sigma^2)$. The λ models the effect of the metal trays, each containing the control and case sample from one site (see Fig 1, main text). A joint maximum likelihood is calculated for both component models to estimate the fixed parameters and the random variance. Residuals were evaluated and met the assumptions of the applied model.

References

AHDB (2021) Nutrient management guide (RB209). Agriculture and Horticulture Development Board, Kenilworth, UK.

Agroscope (2020) Corg: Bestimmung des organisch gebundenen Kohlenstoffs (Corg), Referenzmethode Version 1.2. Zürich: Agroscope, Reckenholz. https://ira.agroscope.ch/de-CH/publication/46276. Accessed on 04.05.2023.

Blüthgen N, Dormann CF, Prati D, Klaus VH, Kleinebecker T, Hölzel N, et al. (2012) A quantitative index of land-use intensity in grasslands: Integrating mowing, grazing and fertilization. Basic and Applied Ecology. 13: 207-220.

Mihelič R, Čop J, Jakše M, F. Štampar F, Majer D, Tojnko S, Vršič S (2010) Smernice za strokovno utemeljeno gnojenje. Ministrstvo za kmetijstvo, gozdarstvo in prehrano, Narodna in univerzitetna knjižnica, Ljubljana, Slovenia.

Richner W, Flisch R, Mayer J, Schleger P, Zähner M, Menzi H (2017) Eigenschaften und Anwendung von Düngern. In: Sinaj S, Richner W, editors. Grundlagen für die Düngung landwirtschaftlicher Kulturen in der Schweiz. Agrarforschung Schweiz 8. p. 1-24.

Totterdell S, Roberts EH (1980) Characteristics of alternating temperatures which stimulate loss of dormancy in seeds of *Rumex obtusifolius* L. and *Rumex crispus* L. Plant, Cell & Environment. 3: 3-12.
