# Supplementary material for: Can the soil seed bank of Rumex obtusifolius in productive grasslands be explained by management and soil properties?
Source: PLoS One. 2023 Jun 2;18(6):e0286760. doi: 10.1371/journal.pone.0286760 (PMC10237634; doi:10.1371/journal.pone.0286760)
Supplement: S1 Table — (PDF) [file pone.0286760.s002.pdf]

S1 Table. General grassland characteristics at the three countries Switzerland (CH), Slovenia (SI), and United Kingdom (UK), where parcels with high and very low density of *Rumex obtusifolius* were investigated to determine the soil seed bank of the species.

		CH	SI	UK
Altitude (m a.s.l.):	range	398 – 967	187 – 678	26 – 231
	median	689	321	120
Defoliation frequency‡: median		5	3	6
Two most dominant species		*Lolium perenne* L.<br>*Trifolium repens* L.	*Lolium perenne* L.<br>*Dactylis glomerata* L.	*Lolium perenne* L.<br>*Holcus lanatus* L.
Sampling time		May – Oct. 2019	Jan. – Dez. 2019	May – Sep. 2020

‡events yr^{-1}
