# Supplementary material for: Can the soil seed bank of Rumex obtusifolius in productive grasslands be explained by management and soil properties?
Source: PLoS One. 2023 Jun 2;18(6):e0286760. doi: 10.1371/journal.pone.0286760 (PMC10237634; doi:10.1371/journal.pone.0286760)
Supplement: S2 Table — (PDF) [file pone.0286760.s003.pdf]

S2 Table. Mean (SD) or frequency of management and soil variables for parcels with high density of *Rumex obtusifolius* (Case) and parcels with very low density or no plants of the species (Control) in the three countries Switzerland (CH), Slovenia (SI), and United Kingdom (UK).

Variable	Unit/Category	CH		SI		UK	
		Case	Control	Case	Control	Case	Control
Management							
Management type	Mowing	9	20	15	16	-[a]	-[a]
	Mixed (mowing-grazing)	14	5	3	4	8	8
	Rotational grazing	14	14	1	0	8	9
	Continuous grazing	3	1	1	0	2	1
Land-use intensity	-	1.7 (0.3)	1.7 (0.3)	1.7 (0.5)	1.6 (0.6)	1.6 (0.7)	1.6 (0.6)
Disturbance	No	2	4	17	16	13	14
	Yes	38	36	3	4	5	4
Regulation	No	6	7	0	14	0	4
	Yes	34	33	20	6	18	14
Soil							
P-AAE[b]	mg kg^{-1}	81.3 (68.6)	58.7 (58.0)	31.8 (33.0)	26.1 (33.6)	85.6 (66.4)	45.5 (42.2)
K-AAE[b]	mg kg^{-1}	382.2 (277.3)	290.1 (191.0)	166.8 (91.6)	138.6 (65.3)	364.4 (178.6)	260.4 (97.0)
Mg-AAE[b]	mg kg^{-1}	275.5 (150.8)	299.3 (206.1)	569.1 (488.8)	639.7 (447.9)	192.7 (61.0)	179.5 (59.4)
Ca-AAE[b]	mg kg^{-1}	6867 (6784)	6480 (5827)	4117 (4111)	4826 (3452)	2901 (1022)	2861 (1091)
pH	-	6.5 (0.6)	6.4 (0.6)	6.3 (0.6)	6.5 (0.6)	6.0 (0.4)	5.8 (0.3)
Organic carbon	%	3.8 (1.2)	4.0 (1.1)	3.0 (0.8)	3.2 (0.9)	3.6 (1.0)	4.1 (0.9)
Clay	%	25.0 (8.4)	26.7 (9.9)	25.0 (10.2)	26.2 (9.2)	26.6 (5.3)	31.2 (7.1)
Silt	%	36.3 (7.0)	35.8 (6.9)	45.1 (9.0)	44.9 (8.4)	36.7 (5.6)	37.7 (6.6)
Sand	%	31.8 (14.2)	30.4 (14.2)	24.7 (14.9)	23.4 (11.5)	30.6 (9.5)	24.0 (10.3)

[a]No mowing at UK, [b]Ammonium acetate extraction
