# Supplementary material for: Can the soil seed bank of Rumex obtusifolius in productive grasslands be explained by management and soil properties?
Source: PLoS One. 2023 Jun 2;18(6):e0286760. doi: 10.1371/journal.pone.0286760 (PMC10237634; doi:10.1371/journal.pone.0286760)
Supplement: S3 Table — (PDF) [file pone.0286760.s004.pdf]

S3 Table. Regression coefficients of variables affecting the number of germinated seeds of *Rumex obtusifolius* in case parcels at the three countries Switzerland (CH), Slovenia (SI), and United Kingdom (UK). Estimates are per unit change on the link-scale of generalised linear models with a log-link, and continuous variables were centered around their mean.

a) Model: ~ Country + pH + silt			
Variable	df	Estimate	95% CI
Intercept (CH)	1	6.739	6.362, 7.169
SI	1	-0.294	-0.979, 0.438
UK	1	-0.578	-1.324, 0.213
pH	1	-0.871	-1.393,-0.309
silt	1	-0.038	-0.076, 0.002
b) Model: ~ Country + LUI			
Intercept (CH)	1	6.741	6.361, 7.175
SI	1	-0.321	-1.003, 0.417
UK	1	-0.213	-0.916, 0.557
LUI	1	-0.630	-1.250, 0.020
c) Model: ~ Country + *Rumex*_density[‡]			
Intercept (CH)	1	6.710	6.327, 7.149
SI	1	-0.257	-0.946, 0.489
UK	1	-0.135	-0.856, 0.662
*Rumex*_density	1	0.087	-0.008, 0.193

[‡]One outlier in UK with 26 *R. obtusifolius* plants m^{-2} omitted; inclusion would have resulted in qualitatively same results.
