# Supplementary figures and images for: Can the soil seed bank of Rumex obtusifolius in productive grasslands be explained by management and soil properties?
Source: PLoS One. 2023 Jun 2;18(6):e0286760. doi: 10.1371/journal.pone.0286760 (PMC10237634; doi:10.1371/journal.pone.0286760)

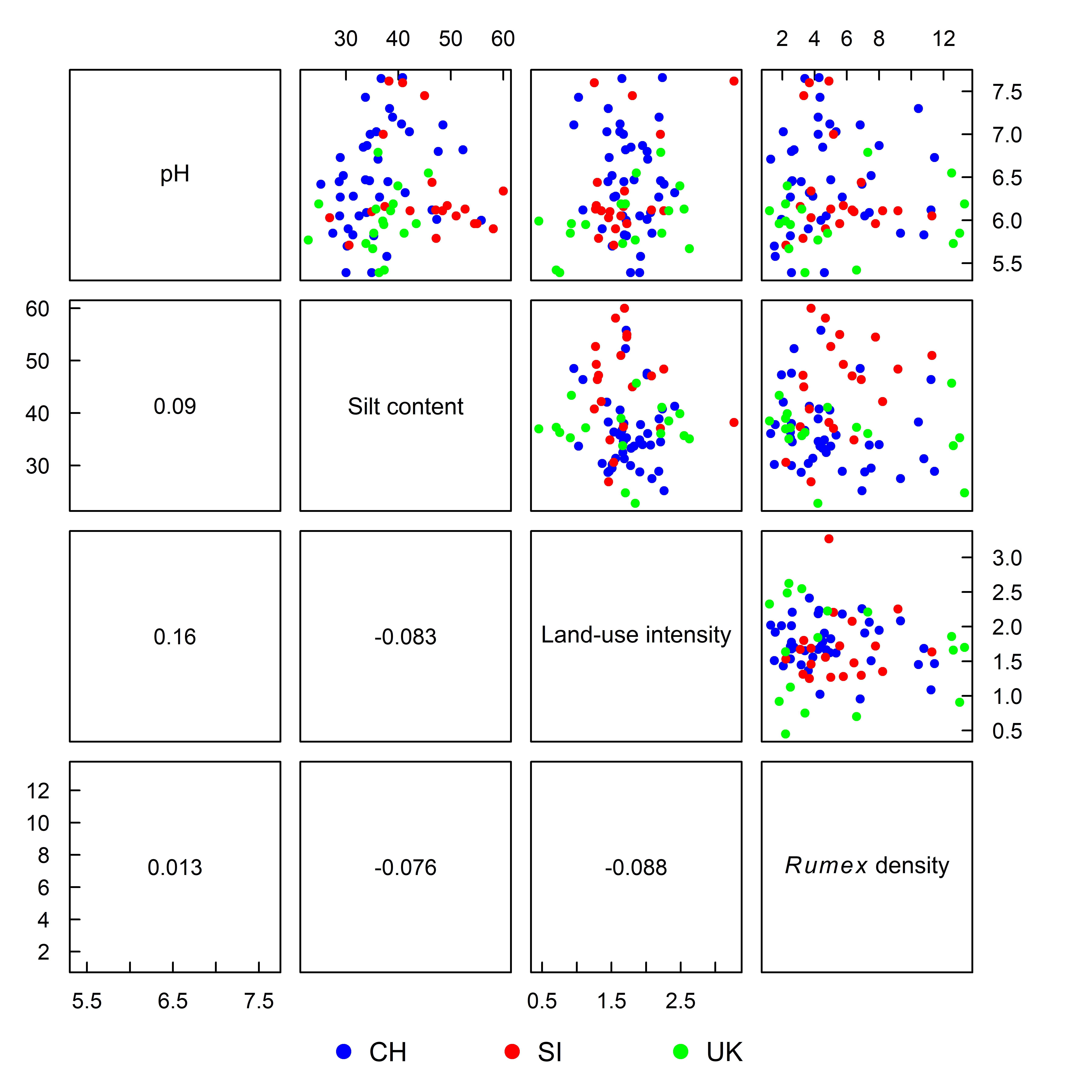

Supplement: S1 Fig — Lower triangle shows Pearson’s coefficients of partial correlation, given country. None of the correlations was significant (p > 0.40 each, with the exception of pH versus land-use intensity: p = 0.16). One outlier in UK with 26 R. obtusifolius plants m-2 was omitted; inclusion would have resulted in qualitatively same results. See S2 Table for units of variables. (JPG) [file pone.0286760.s005.jpg]

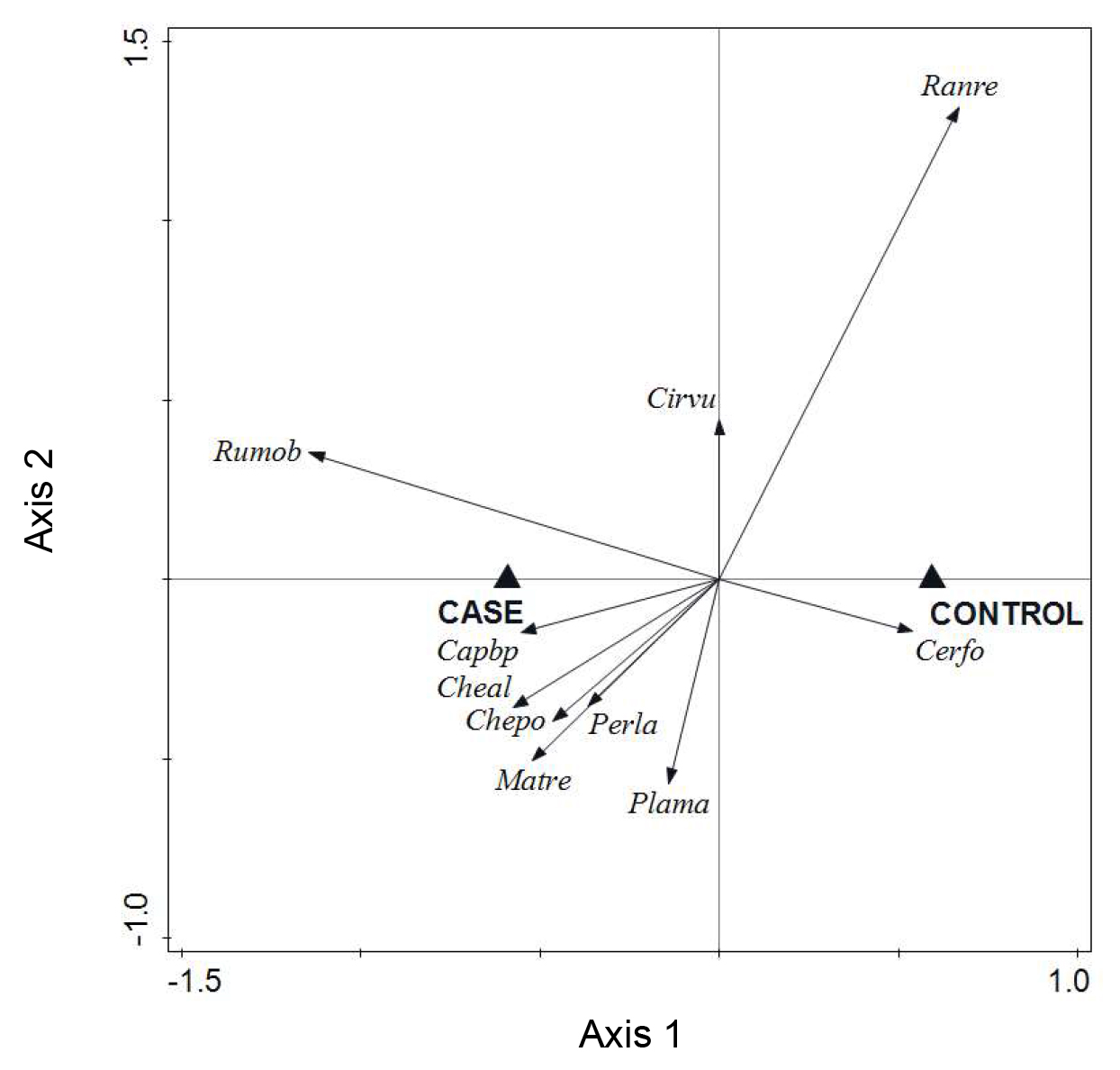

Supplement: S2 Fig — Rumex obtusifolius (Rumob) was associated with a more diverse seed bank dominated by annuals. CASE: ≥ 1 R. obtusifolius plant m-2, CONTROL: ≤ 100 R. obtusifolius plants m-2, Capbp: Capsella bursa-pastoris, Cerfo: Cerastium fontanum, Cheal: Chenopodium album, Chepo: Chenopodium polyspermum, Cirvu: Cirsium vulgare, Matre: Matricaria recutita, Perla: Persicaria lapathifolia, Plama: Plantago major, Ranre: Ranunculus repens. (JPG) [file pone.0286760.s006.jpg]
